# Psychological contract and turnover intention among healthcare assistants in a hospital environment: a multi-center cross-sectional analysis

**DOI:** 10.3389/fpsyg.2025.1517138

**Published:** 2025-04-11

**Authors:** Jing-ping Yang, Feng Xue, Lin Zhang, Qiaoxian Zhang

**Affiliations:** Department of Nursing, The First Affiliated Hospital of Fujian Medical University, Fuzhou, China

**Keywords:** psychological contract, turnover intention, health care assistant, hospital, Chinese

## Abstract

**Aim:**

The aim of this study was to examine the correlation between psychological contract and turnover intention among hospital healthcare assistants.

**Design:**

A multi-center cross-sectional analysis was employed in this study.

**Methods:**

A survey was conducted among healthcare assistants employed at 10 Grade A tertiary hospitals in Fuzhou using a psychological contract scale, turnover intention scale, and demographic questionnaire.

**Results:**

A total of 540 healthcare assistants participated in the study. The turnover intention scores varied from 6 to 22, with an average score of 11.51 ± 3.54. The number of individuals with a very low turnover intention amounts to 40 (7.40%), those with a low turnover intention total 302 (55.93%), individuals with moderate turnover intention numbered 195 (36.11%), and those with a high turnover intention are 3 (0.56%).The psychological contract scores among healthcare assistants ranged from 42 to 195, with an average score of 153.90 ± 33.18. A hierarchical multiple regression analysis was performed and psychological contract increased the explanatory power of the turnover intention regression model by 38.4%.

**Conclusion:**

Maintaining a high level of psychological contract is beneficial for reducing the turnover intention of healthcare assistants. Hospital managers can adopt certain measures from the perspective of psychological contract to stabilize the team of healthcare assistants.

## 1 Introduction

The global population is rapidly aging, posing formidable challenges to healthcare systems. By 2050, 16% of the world’s population will be over 65, up from 10% in 2022 (The United Nations, 2024). China mirrors this trend: its elderly population surged from 88 million (2000) to 287 million (2022) (Chen et al., 2022; National Bureau of Statistics, 2024), with total population projected to peak at approximately 1.4 billion around 2035 before declining (Chen et al., 2022). Healthcare systems must urgently adapt to rising demands—particularly for chronic disease management, long-term care, and end-of-life support. This signifies a substantial rise in the demand for nursing personnel; however, it is estimated that China faces a shortage of nearly 4 million nurses (The Central People’s Government of the people’s Republic of China, 2021; The Central People’s Government of the people’s Republic of China, 2024). Furthermore, family structures in China have evolved significantly. Single-person, one-generation and two-generation households are increasingly common while three-generation households have declined (Yang and Chen, 2019). This demographic shift in household composition has significantly reduced the availability of familial caregivers, thereby significantly diminishing informal caregiving capacity and increasing reliance on nursing services. Moreover, as living standards rise, there is an increasing demand for comprehensive, multi-level, and personalized nursing services, which further exacerbates the existing shortage of nursing professionals in China.

To bridge this workforce gap, the National Health Commission has positioned healthcare assistants as a strategic supplement to nurses (The Central People’s Government of the people’s Republic of China, 2022; The Central People’s Government of the people’s Republic of China, 2019). As part of the medical auxiliary workforce, healthcare assistants can partially alleviate the workload of nurses (Kroezen et al., 2018). By enhancing their training and oversight (The Central People’s Government of the people’s Republic of China, 2019), this policy not only expands service accessibility but also elevates overall care quality-an essential step toward sustainable elderly care systems. Healthcare assistants have become a common presence in developed countries in the West, where many nations have well-established legal and institutional safeguards. In recent years, some Chinese hospitals have implemented policies such as standardized training programs and tiered management systems for healthcare assistants. However, the management of healthcare assistants in China is fraught with numerous problems, including irregular training, inadequate supervision, and an imperfect social security system, which have contributed to the instability of the healthcare assistant workforce (Zhang et al., 2019; He, 2020; Wang, 2022). As outlined by Wu (2007) in the employee turnover model, psychological contract serve as the intrinsic foundation of organizational commitment and job satisfaction, and they have a significant correlation with turnover intention. Therefore, it is of great significance to study the impact of the psychological contract on turnover intention for stabilizing healthcare assistants.

While existing studies have provided valuable insights into the psychological contract of healthcare assistants in long-term care settings (Wang et al., 2021; Rao, 2017; Li et al., 2016), the unique of acute care environments remain unexplored. Specifically, within China’s rapidly evolving healthcare landscape, this aspect remains underexamined. Unlike nursing homes, hospitals are characterized by higher workloads, interdisciplinary collaboration, and acute care demands. Due to these different factors, the psychological contract of healthcare assistants may also present different situations, but this has not been explored. This study aims to explore the correlation between the psychological contract and turnover intention among healthcare assistants through surveys and analyses conducted at Grade A tertiary hospitals in Fuzhou, to bridge this research gap. It seeks to offer scientific management strategies for medical institutions to achieve the goals of stabilizing the healthcare assistants workforce and enhancing the quality of healthcare services.

## 2 Method

### 2.1 Study design, setting and participants

Fujian Province, a pioneer in piloting‘No Company Ward’project, exemplifies efforts to professionalize healthcare assistants (Fujian Provincial Health Commission, 2022). This multicenter cross-sectional study was conducted in Fuzhou, the capital of Fujian Province, from January to March 2023. Fuzhou has a population of about 8.5 million. A cluster sampling strategy was utilized, and participants were methodically selected from ten Grade A tertiary hospitals across the city. Grade A tertiary hospitals represent China’s highest care standards, where healthcare assistants face intensified pressures. Studying this cohort ensures insights into high-stress environments. Inclusion criteria: Those who have participated in healthcare assistant practice-related training and passed the assessment; those employed by hospitals or third-party organizations; those with a work experience of ≥3 months in clinical front-line healthcare assistant positions; those without communication and understanding barriers. It is important to note that no compensation was provided to participants for their participation in the study.

### 2.2 Measures

The Psychological Contract Scale was employed to assess the level of psychological contract among healthcare assistants (Li, 2006). The 39-item Psychological Contract Scale includes two subscales: Organizational Responsibility and Employee Responsibility. Each subscale is further subdivided into three dimensions: Normative Responsibility, Interpersonal Responsibility, and Developmental Responsibility. The scale employs a 5-point Likert scale for scoring, with responses ranging from “Strongly Disagree” to “Strongly Agree,” corresponding to scores from 1 to 5, respectively. The total score ranges from 39 to 195, with higher scores indicating a greater extent of alignment with the psychological contract. In the present study, the Cronbach’s alpha coefficient for the scale was 0.978, for the Organizational Responsibility subscale it was 0.955, and for the Employee Responsibility subscale it was 0.981.

The Chinese version of the Turnover Intention Scale was used to measure the turnover intention among healthcare assistants (Li and Li, 2000). This scale comprises 6 items, categorized into 3 dimensions. Items 1 and 6 correspond to Dimension I of turnover intention, reflecting the likelihood of an individual departing from their current job. Items 2 and 3 correspond to Dimension II of turnover intention, indicating contemplation of seeking alternative employment. Items 4 and 5 correspond to Dimension III of turnover intention, suggesting the probability of obtaining external job opportunities. Responses to questions 1–3 are scored as follows: “Never” as 1, “Rarely” as 2, “Occasionally” as 3, and “Frequently” as 4. Responses to questions 4–5 are scored: “Extremely Unlikely” as 1, “Unlikely” as 2, “Possible” as 3, with “Extremely Possible” also scoring as 4. The sixth item is scored with: “Definitely Not” as 1, “Possibly Not” as 2, “Possibly” as 3, and “Definitely” as 4. A higher aggregate score signifies a stronger turnover intention. Within the scope of this study, the mean total score of turnover intention was ≤1 for very low turnover intention, >1 and ≤ 2 for low turnover intention, >2 and ≤ 3 for moderate turnover intention, and > 3 for high turnover intention. The Cronbach’s alpha coefficient for the scale in this study was 0.827.

In addition to the above, the study also collected socio-demographic data on the healthcare assistants. The content of the socio-demographic data was determined through a combination of literature review and group discussions. The collected data included the following: gender, age, educational level, monthly income, length of service, whether they were assigned to a specific department, daily working hours, whether their workplace provided training, and whether their family and colleagues supported their work.

### 2.3 Data collection procedure

The data were collected by a research assistant (RA). To assess the logistic issues and feasibility of the study, 10 healthcare assistants were recruited for the pilot study. No significant issues were encountered during this phase. The RA explained the details, purpose, and procedure of the study to the eligible healthcare assistants. Participants who provided informed written consent were then asked to complete the questionnaires in a quiet room, free from interruptions by others. The research assistant remained nearby to address participants’ questions and collect completed questionnaires.

### 2.4 Data analysis

Data were analyzed using IBM SPSS Statistics version 25.0 (IBM Corporation, Armonk, NY, USA). Descriptive statistics were employed to summarize social-demographic and study variables, including frequencies (percentages), means, and standard deviations. For one-way analysis, t-tests or analysis of variance were utilized. The association between the psychological contract and turnover intention was assessed through Pearson correlation analysis. The impact of the psychological contract on healthcare assistants’ turnover intention was further explored using hierarchical regression analysis. A significance level of *p* < 0.05 was considered statistically significant.

## 3 Results

A total of 567 healthcare assistants were invited to participate in the study, of whom seven chose not to participate. The primary reasons for their refusal were being too busy, tiredness and reluctance to disclose personal information. Of the 560 healthcare assistants who agreed to participate, 20 did not complete the full assessment, and were subsequently excluded from the analysis. Consequently, a total of 540 healthcare assistants (response rate = 95.24%) completed the questionnaires.

The turnover intention scores among healthcare assistants varied from 6 to 22, with an average score of 11.51 ± 3.54. The average scores for the three dimensions were 3.44 ± 1.33, 3.39 ± 1.48, and 4.69 ± 1.50, respectively. The number of individuals with a very low turnover intention amounts to 40 (7.40%), those with a low turnover intention total 302 (55.93%), individuals with a moderate turnover intention number 195 (36.11%), and those with a high turnover intention are 3 (0.56%).

The psychological contract scores among healthcare assistants ranged from 42 to 195, with an average score of 153.90 ± 33.18. The scores for the organizational responsibility subscale ranged from 24 to 105, with an average score of 80.78 ± 17.43, including an average of 18.16 ± 4.22 for normative responsibility, 34.27 ± 8.14 for interpersonal responsibility, and 28.35 ± 6.65 for development responsibility. The employee responsibility subscale scores ranged from 18 to 90, with an average score of 73.12 ± 16.74, including an average of 24.45 ± 5.62 for normative responsibility, 20.67 ± 4.60 for interpersonal responsibility, and 28.00 ± 6.90 for development responsibility.

Table 1 displays the socio-demographic characteristics of the participants and the five variables that were significantly correlated with turnover intention, which were included in the multivariate regression model for turnover intention. Table 1 also includes four variables significantly correlated with psychological contract. The fixed department work, unit organizes training, family support for work, and colleague support for work are all related to the turnover intention and psychological contract of healthcare assistants.

**Table 1:** Differences in the turnover intention among various socio-demographic (n = 540).

Characteristics	Number	Turnover Intention	t/F	*p*	psychological contract	t/F	*p*
Gender			t = 0.750	0.454		0.337	0.736
Male	260	11.40 ± 3.47			153.40 ± 34.63		
Female	280	11.63 ± 3.61			154.36 ± 31.84		
Age (years)			*F* = 0.168	0.845		0.641	0.527
≤40	28	11.57 ± 3.55			148.75 ± 39.92		
41 ~ 50	225	11.61 ± 3.49			152.93 ± 33.84		
>50	287	11.43 ± 3.59			155.16 ± 31.98		
Educational level			t = 0.041	0.967		1.922	0.055
Primary and below	238	11.69 ± 3.60			147.84 ± 36.46		
Secondary and above	237	11.68 ± 3.84			153.87 ± 31.64		
Length of service (year)			*F* = 4.920	0.008		2.852	0.059
≤5	109	11.13 ± 3.48			151.75 ± 38.18		
5 ~ 10	196	11.08 ± 3.50			158.39 ± 29.07		
>10	235	12.06 ± 3.55			151.45 ± 33.66		
Monthly Income (RMB)			*F* = 0.316	0.729		0.639	0.528
2,000 ~ 4,000	34	11.94 ± 3.68			147.71 ± 35.88		
4,000 ~ 6,000	342	11.45 ± 3.50			154.44 ± 32.69		
6,000~	164	11.56 ± 3.61			154.04 ± 33.71		
Fixed Department Work			t = 4.904	<0.001		5.920	<0.001
Yes	300	10.86 ± 3.31			161.46 ± 27.57		
No	240	12.33 ± 3.66			144.45 ± 37.03		
Daily working hours			t = −0.056	0.956		0.110	0.912
8-12 h	210	11.67 ± 3.83			151.04 ± 32.03		
>12 h	265	11.69 ± 3.63			150.69 ± 35.94		
Unit Organizes Training			t = 5.330	<0.001		4.894	<0.001
Yes	222	10.74 ± 3.47			158.81 ± 31.55		
No	253	12.51 ± 3.74			143.86 ± 35.02		
Family Support for Work			t = 7.060	<0.001		7.686	<0.001
Yes	224	10.47 ± 3.52			162.72 ± 26.68		
No	251	12.76 ± 3.56			140.25 ± 36.72		
Colleague Support for Work			t = 8.118	<0.001		7.631	<0.001
Yes	268	10.54 ± 3.47			161.04 ± 28.99		
No	207	13.16 ± 3.51			137.65 ± 36.00		

As indicated in Table 2, all dimensions of the psychological contract were significantly linked to turnover intention and were thus included in the multivariate regression model for turnover intention.

**Table 2:** The correlation between the psychological contract and the turnover intention among healthcare assistants(r).

Variables	Turnover Intention	Dimension I	Dimension II	Dimension III
Psychological Contract	−0.615**	−0.564**	−0.594**	−0.367**
Organizational Normative Responsibility	−0.618**	−0.535**	−0.565**	−0.427**
Organizational Interpersonal Responsibility	−0.623**	−0.558**	−0.588**	−0.396**
Organizational Development Responsibility	−0.562**	−0.525**	−0.551**	−0.319**
Employee Normative Responsibility	−0.522**	−0.491**	−0.517**	−0.287**
Employee Interpersonal Responsibility	−0.493**	−0.459**	−0.480**	−0.285**
Employee Development Responsibility	−0.551**	−0.517**	−0.544**	−0.306**

**p < 0.001.

A hierarchical multiple regression analysis was performed (Table 3). The results indicate that colleague support, length of service, and psychological contract are influencing factors on the turnover intention of healthcare assistants. As illustrated in Table 3, The results of the first layer regression indicate that three variables are related to turnover intention, explaining 10.4% of the variance. However, the explanatory power for the turnover intention of healthcare assistants increased to 48.8% after incorporating psychological contract.

**Table 3:** Predictors of turnover intention among healthcare assistants (*n* = 540).

Variables	First Layer (socio-demographic)				Second Layer (psychological contract)			
	B	Standard error-B	t	*p*	B	Standard error-B	t	*p*
Fixed Department Work^a^	−1.345	0.298	−4.513	<0.001	−0.279	0.233	−1.197	0.232
Family Support for Work^b^	−0.092	0.342	−0.269	0.788	0.162	0.259	0.623	0.534
Colleague Support for Work^c^	−0.863	0.372	−2.320	0.021	−0.611	0.284	−2.148	0.032
Unit Organizes Training^d^	0.325	0.266	1.220	0.223	−0.050	0.206	−0.243	0.808
Length of service (year)								
≤5	Ref.							
5 ~ 10	0.061	0.403	0.152	0.879	0.397	0.306	1.297	0.195
>10	1.046	0.391	2.675	0.008	0.761	0.298	2.556	0.011
Organizational Normative Responsibility					−0.274	0.052	−5.264	<0.001
Organizational Interpersonal Responsibility					−0.122	0.036	−3.376	0.001
Organizational Development Responsibility					−0.541	0.109	−4.965	<0.001
Employee Normative Responsibility					0.284	0.072	3.968	<0.001
Employee Interpersonal Responsibility					0.336	0.080	4.209	<0.001
Employee Development Responsibility					0.053	0.083	0.643	0.521
*R* ^2^	0.114				0.499			
Adjust *R*^2^	0.104				0.488			
F	11.447				43.811			
*p*	<0.001				<0.001			

a Fixed Department Work: no = 0, yes = 1.

b Family Support for Work: no = 0, yes = 1.

c Colleague Support for Work: no = 0, yes = 1.

d Unit Organizes Training: no = 0, yes = 1.

## 4 Discussion

By examining psychological contract in high-pressure tertiary hospitals, this study addresses a critical gap in understanding turnover drivers among healthcare assistants, a population often overlooked in prior research. Turnover intention refers to the extent to which an individual desires to leave their current place of employment in search of alternative job opportunities (Chen and Wang, 2019). Studies have shown that turnover intention can predict actual turnover behaviors, suggesting that exploring turnover intention may provide hospital managers with new perspectives for maintaining a stable workforce. The results of this study indicate that the turnover intention among healthcare assistants is at a relatively low level, which contrasts with the findings of Li et al. (2016). This discrepancy may be attributed to the longer tenure of participants in the present study. Additionally, differences in the professional institutions of healthcare assistants across the two studies could contribute to this variation. The study reveals that dimension III of turnover intention scored the highest, suggesting a greater likelihood of individuals securing employment outside their current environment. This finding aligns with the current shortage of healthcare assistants in healthcare institutions. However, in China, many healthcare assistants are from the lower socioeconomic strata of rural society, characterized by an older age, lower educational levels, and insufficient health literacy (Chen and Zhang, 2022). Despite the relatively favorable working conditions and economic income provided by medical institutions, it is still challenging for healthcare assistants to self-assess their ability to find a stable job, which explains the lower scores for dimension I and dimension II of the turnover intention.

The results of this study indicate that the psychological contract level among healthcare assistants is lower than that reported by Wang et al. (2021), potentially due to regional disparities. Having a fixed department, organizational training by the unit, family support for work, and coworker support for work are all conducive to the psychological contract of caregivers, as these factors are consistent with the connotations of the psychological contract. Within the organizational responsibility subscale, the highest scores were observed in the organizational interpersonal responsibility dimension, indicating that medical institutions provide a relatively harmonious interpersonal environment and a positive work atmosphere. The lowest scores in organizational normative responsibility correspond with previous findings (Wang et al., 2021; Rao, 2017), highlighting unmet expectations among healthcare assistants and reflecting systemic gaps in China’s institutional frameworks for this workforce. Specifically, the lack of standardized policies—such as comprehensive social insurance and transparent career progression pathways—may exacerbate perceptions of instability. To reinforce the psychological contract, policymakers and hospital administrators should prioritize implementing robust regulations that address these structural deficiencies.

In the employee responsibility subscale, the highest score was observed for employee development responsibility, while the lowest score was detected in the employee interpersonal responsibility dimension. This disparity could be attributed to the more rigorous professional qualification requirements for healthcare assistants in medical institutions and the complex interpersonal relationships within these settings, characterized by competitive positional struggles. This competitive environment may contribute to collegial strain. Therefore, it is suggested that the relevant authorities should establish and refine regulations and systems for healthcare assistants, foster a positive employment environment, and improve the career development paths for healthcare assistants.

The study findings indicate that all dimensions of the psychological contract among healthcare assistants are inversely related to turnover intention and the hierarchical regression analysis reveals that incorporating the dimensions of the psychological contract explained an additional 38.4% of the variance in the regression model, underscores the centrality of psychological contract in turnover dynamics. Organizational developmental responsibility contributed most to reducing turnover intention, suggesting career growth opportunities are pivotal—a finding aligning with Rodwell & Gulyas (Rodwell and Gulyas, 2013). However, potential collinearity between dimensions and unmeasured variables necessitates caution in causal interpretation.

In the regression model, each dimension of the organizational responsibility scale exhibits a negative correlation with healthcare assistants’ turnover intention. In other words, the greater the level of responsibility perceived by employees towards their employer, the lower their inclination to leave. This points to the necessity for employers to create a structured salary system, provide equitable welfare support, and maintain a positive employment environment. Furthermore, it is imperative for managers to recognize the vital role that standardized training plays in ensuring patient safety (Blay and Roche, 2020). This can be accomplished by instituting a tiered management structure, assigning nursing tasks in accordance with the capabilities of the healthcare assistants, and offering comprehensive guidance and training for the knowledge and skills necessary for their roles. Emphasis should be placed on work quality and patient satisfaction as primary metrics, conducting rigorous quality oversight and evaluation to further standardize service conduct and enhance service quality. In terms of employee responsibility scale, normative responsibility and interpersonal responsibility exhibit a positive correlation with healthcare assistants’ turnover intention, which may reflect excessive role demands. When healthcare assistants perceive excessive demands to fulfill organizational expectations beyond their formal duties, job strain may escalate, paradoxically increasing resignation risks.

While most demographic variables showed no significant correlation with turnover intention in regression models, the results indicated that healthcare assistants with more than 10 years of service reported higher turnover intention compared to those with 5 years or less. This suggests that long-term employees may experience cumulative dissatisfaction, potentially reflecting systemic barriers in China’s healthcare assistant career pathways. Long-serving assistants often face stagnant roles without promotion opportunities, leading to accumulated job strain.

Healthcare assistants should enhance professional accountability through continuous learning, aligning personal growth with organizational goals. Nevertheless, considering the constraints faced by healthcare assistants regarding age and educational background, employers must innovate in training and motivational strategies for these professionals. They should foster a robust culture of professional growth, cultivate a collaborative and supportive work environment, establish a positive psychological contract with healthcare assistants, and build a mutually beneficial employment relationship to ensure the sustained, high-quality development of the healthcare assistant workforce.

The high variance explained by the regression model underscores the critical role of psychological contract, yet it is essential to acknowledge that unobserved factors might interact with the studied variables. Further longitudinal or mixed-methods research could disentangle these complex dynamics. It is important to note that this study was conducted exclusively in Grade A tertiary hospitals in Fuzhou, which may limit the generalizability of the findings to other regions or healthcare settings. Future studies should expand the geographic scope and include diverse types of medical facilities to validate these results across different contexts.

## 5 Conclusion and limitations

These findings contribute to the broader literature on psychological contracts by highlighting how contextual factors in the healthcare sector—such as regulatory gaps, socio-economic conditions, and institutional culture—moderate the relationship between psychological contract fulfillment and turnover intention. Hospital managers should assess and align mutual expectations and obligations to stabilize the workforce. They should also focus on improving training and regulation for healthcare assistants, and establish a comprehensive career protection system. Healthcare assistants, in turn, should fulfill their responsibilities, enhance service skills, and collaborate closely with employers to maintain a strong psychological contract, ensuring workforce stability.

The research was limited to 10 Grade A hospitals in Fuzhou due to resource and time constraints, resulting in limited geographical representation. Future studies could expand the scope and include a mix of quantitative and qualitative methods to better understand the impact of psychological contract on healthcare assistants’ turnover intentions. This cross-sectional study does not establish causality, so longitudinal studies could confirm the temporal relationship between contract violation and turnover behavior. To mitigate potential biases from self-reported data, future research could combine objective indicators with qualitative interviews for methodological triangulation.

## Data Availability

The raw data supporting the conclusions of this article will be made available by the authors, without undue reservation.
